# Taylor on Genito-Urinary and Venereal Diseases and Syphilis

**Published:** 1901-08

**Authors:** 


TAYLOR ON GENITO-URINARY AND VENEREAL DISEASES AND SYPHILIS.—The pathology and treatment of genito-urinary and Venereal diseases and syphilis. By Robert W. Taylor, A. M., M. D., Clinical Professor of Venereal Diseases in the College of Physicians and Surgeons, New York. New (second) edition. In one very handsome octavo volume of 720 pages, with 135 engravings and 27 full-page plates in colors and monotone. Cloth, $5.00, net; leather, $6.00, net. Lea Brothers & Co., Publishers, Philadelphia and New York.

The author of this work brought out a book last year entitled "A Practical Treatise on Sexual Disorders of the Male and Female." The present work is an enlargement and revision of the first, and includes genito-urinary diseases. This, the present volume, makes a most complete and comprehensive exposition of these closely related subjects. This work will be appreciated by the profession. It is thoroughly practical. T. J. B.

